# Supplementary material for: Mathematical modeling of multiple pathways in colorectal carcinogenesis using dynamical systems with Kronecker structure
Source: PLoS Comput Biol. 2021 May 18;17(5):e1008970. doi: 10.1371/journal.pcbi.1008970 (PMC8162698; doi:10.1371/journal.pcbi.1008970)
Supplement: S1 Appendix — This includes basic notions from graph theory, graph products, the Kronecker sum of matrices, linear dynamical systems and their solution. (PDF) [file pcbi.1008970.s001.pdf]

Supporting Information S1 Appendix
Mathematical Background

Mathematical Modeling of Multiple Pathways in Colorectal Carcinogenesis using Dynamical Systems with Kronecker Structure

Saskia Haupt[1,2*], Alexander Zeilmann[3], Aysel Ahadova[4], Hendrik Bläker[5], Magnus von Knebel Doeberitz[4], Matthias Kloor[4], Vincent Heuveline[1,2]

1 Engineering Mathematics and Computing Lab (EMCL), Interdisciplinary Center for Scientific Computing (IWR), Heidelberg University, Heidelberg, Germany
2 Data Mining and Uncertainty Quantification (DMQ), Heidelberg Institute for Theoretical Studies (HITS), Heidelberg, Germany
3 Image and Pattern Analysis Group (IPA), Heidelberg University, Heidelberg, Germany
4 Department of Applied Tumor Biology (ATB), Institute of Pathology, University Hospital Heidelberg, Heidelberg, Germany
5 Institute of Pathology, University Hospital Leipzig, Leipzig, Germany

* saskia.haupt@uni-heidelberg.de

We will give a short introduction to the mathematical background of our model. We start with a few basic notions from graph theory, before we state three assumptions about the properties a combination of several processes should have. The assumptions are then related to graph products and the Kronecker sum of matrices. Finally, we consider linear dynamical systems and their solution.

1 A Primer on Graph Theory

Modeling evolutionary processes can often be done with the help of graphs $\mathcal{G} = (\mathcal{V}, \mathcal{E})$, i.e., as set $\mathcal{V}$ of states (vertices) which are combined with edges $\mathcal{E}$ if a progression between the states is possible.

We represent the graph as an adjacency matrix A, that is, a matrix which has as many rows and columns as there are vertices in the graph. The entry $a_{i,j}$ at position (i, j) of the matrix A indicates whether there is no connection ($a_{i,j} = 0$), a weak relationship ($a_{i,j}$ is small) or a strong bond ($a_{i,j}$ is large) between the vertices i and j. The value $a_{i,j}$ is often referred to as the weight of the edge ij. Vertices i and j which are connected by an edge are called adjacent and are denoted by $i \sim j$. Vertices may also be connected to themselves. In this case, the edge is called a self-loop.

It is also possible to indicate directed edges, by letting $a_{i,j}$ (weight of the edge from i to j) differ from $a_{j,i}$ (weight of the edge from j to i). A directed graph with no directed cycles is called a directed acyclic graph (DAG). The vertices of a DAG can be ordered such that the adjacency matrix is an upper triangular matrix.

2 Combining Processes and the Cartesian Product of Graphs

Now assume that we have two processes, e.g., the occurrence of mutations in two different genes of the same cell, which we want to combine and represent as one single process. For this combination we have the following key assumptions:

Existence of States The states in the combined process should exactly be all possible combinations of states from the underlying processes.

Edge Connectivity We require that in the combined model only one of the processes can evolve to the next state at any point in time.

Independence of the Processes We require that the processes are independent of each other.

In the context of the example above, we interpret from the first assumption that all combinations of mutations in the different genes are possible (i.e., there are no mutations that prevent other mutations) and that there are no additional states. This also implies that the order in which mutations are accumulated is ignored.

The edge connectivity assumption states that no two mutations in different genes can occur at the exactly same point in time.

The third assumption entails that a mutation in one gene does not change the mutation probability in another gene. While the independence is a strong requirement, it makes the mathematical analysis more amenable by reducing the number of parameters and improving the model structure. From a medical point of view, we consider this independence for all mutational processes where there are no data currently available about mutational dependencies or where data suggests independence. Whenever there are data available, we introduce this dependency in our model (see also Section 3.1.5 in the main text).

This mathematical approach allows for the implementation of further genetic dependencies as soon as novel experimental data are generated. As the matrices are built in a structured way using the Kronecker structure, adding novel dependencies will not lead to a bottleneck with respect to computational costs.

Consider two processes that are represented by the graphs $\mathcal{G}_1 = (\mathcal{V}_1, \mathcal{E}_1)$ and $\mathcal{G}_2 = (\mathcal{V}_2, \mathcal{E}_2)$. The combined process, which satisfies our key assumptions, is then represented by a graph $\mathcal{G} = (\mathcal{V}, \mathcal{E})$, that is given by the Cartesian product [1], denoted by a small square $\square$, of the graphs $\mathcal{G}_1$ and $\mathcal{G}_2$

$$\mathcal{G} = \mathcal{G}_1 \,\square\, \mathcal{G}_2. \tag{S1-1}$$

The set of vertices $\mathcal{V}$ of the Cartesian graph product is given by the Cartesian product $\mathcal{V} = \mathcal{V}_1 \times \mathcal{V}_2$ of the vertex sets $\mathcal{V}_1$ and $\mathcal{V}_2$. This means the vertex set $\mathcal{V}$ consists of all possible combinations of vertices from the first graph with vertices from the second graph

$$\mathcal{V} = \{(v_1, v_2) | v_1 \in \mathcal{V}_1 \text{ and } v_2 \in \mathcal{V}_2\}\,. \tag{S1-2}$$

In other words, the first requirement is satisfied.

The edge set $\mathcal{E}$ is made up of edges of the forms

$$(v_1, v_2) \sim (w_1, v_2)\,, \quad (v_1, v_2) \sim (v_1, w_2)\,, \tag{S1-3}$$

where the vertices $v_1, w_1 \in \mathcal{V}_1$ are adjacent in the graph $\mathcal{G}_1$ and similarly for $v_2, w_2 \in \mathcal{V}_2$. This means that we connect the states in our combined process such that

each edge corresponds to a single edge in exactly one of the underlying processes. From this we establish that our second assumption is also fulfilled.

As each edge in $\mathcal{E}$ corresponds to exactly one of the edges in $\mathcal{E}_1 \cup \mathcal{E}_2$, we can transfer the edge weights from the graphs $\mathcal{G}_1$ and $\mathcal{G}_2$ to $\mathcal{G}$. From this, we conclude the satisfaction of the independence assumption.

We can extend this combination to more than two processes by iteratively applying the definition to two of the processes.

To relate the Cartesian product of graphs to their adjacency matrices, we first need to consider the Kronecker product and sum of two matrices.

3 Kronecker Product and Sum

The Kronecker product $A \otimes B \in \mathbb{R}^{mp \times nq}$ of two matrices $A \in \mathbb{R}^{m \times n}$ and $B \in \mathbb{R}^{p \times q}$ is defined [2, 3] by the block matrix

$$A \otimes B = \begin{pmatrix} a_{1,1}B & \dots & a_{1,n}B \\ \vdots & \ddots & \vdots \\ a_{m,1}B & \dots & a_{m,n}B \end{pmatrix}. \tag{S1-4}$$

For square matrices $A \in \mathbb{R}^{n \times n}$ and $B \in \mathbb{R}^{m \times m}$, we further define the Kronecker sum

$$A \oplus B = A \otimes I_m + I_n \otimes B \quad \in \mathbb{R}^{mn \times mn}, \tag{S1-5}$$

where I_m (resp. I_n) denotes the identity matrix of size $m \times m$ (resp. $n \times n$).

The Kronecker product and sum possess several favorable properties, among which is the compatibility with the matrix transpose

$$(A \otimes B)^\top = A^\top \otimes B^\top, (A \oplus B)^\top = A^\top \oplus B^\top. \tag{S1-6}$$

Let A_1 and A_2 be the adjacency matrices of the graphs $\mathcal{G}_1$ and $\mathcal{G}_2$. The adjacency matrix of the Cartesian Product $\mathcal{G}_1 \,\square\, \mathcal{G}_2$ is given by the Kronecker sum $A_1 \oplus A_2$ [4].

This connection between the Cartesian product of graphs and the Kronecker sum is

visualized in Fig A.

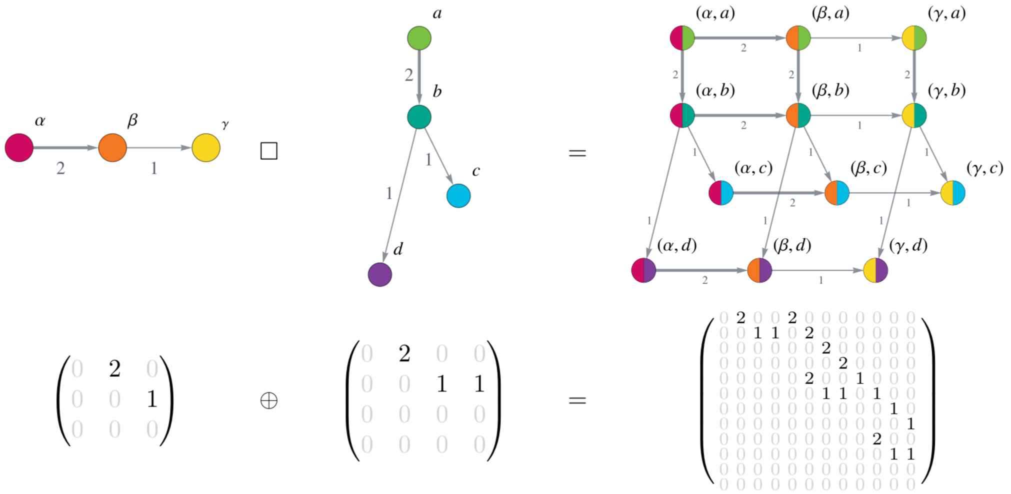

Fig A. Cartesian Product of Graphs. The upper row shows two graphs and their Cartesian product. Notice how each vertex (α, β, γ) of the first graph is combined with each vertex (a, b, c, d) from the second graph, yielding a total of 12 $(= 3 \cdot 4)$ vertices in the Cartesian graph product. The edge weights (indicated by numbers next to the edges and the edge thickness) of the graphs on the left and middle transfer to the corresponding edges in the product graph. The bottom row displays the adjacency matrices corresponding to the graphs in the upper row as an equation involving the Kronecker sum of the matrices.

The Kronecker product is not commutative, i.e., in general we have $A_1 \oplus A_2 \neq A_2 \oplus A_1$. Accordingly, the graph products $\mathcal{G}_1 \square \mathcal{G}_2$ and $\mathcal{G}_2 \square \mathcal{G}_1$ are not equal to each other. However, as they are isomorphic to each other, we are free to choose an ordering of the matrices as long as we use the same ordering in all computations.

4 Linear Dynamical Systems

An important and highly used class of equations for dynamical systems in mathematical modeling are linear differential equations of the form

$$\dot{x}(t) = Ax(t), x(0) = x_0, \tag{S1-7}$$

where $A \in \mathbb{R}^{n \times n}$ is a matrix (later chosen to be the adjacency matrix of a graph), which is constant with respect to time, and $x_0 \in \mathbb{R}^n$ is a vector representing the initial value.

Linear differential equations have a unique solution [5, p. 60], which is given by

$$x(t) = \operatorname{expm}(tA)\, x_0 \quad \forall t \in \mathbb{R}, \tag{S1-8}$$

where $\operatorname{expm}(A)$ describes the matrix exponential of the matrix A, which is defined by

$$\operatorname{expm}\colon \mathbb{R}^{n\times n} \longrightarrow \mathbb{R}^{n\times n}, \quad A \longmapsto \sum_{k=0}^{\infty} \frac{1}{k!} A^k. \tag{S1-9}$$

Computing the matrix exponential [6] can be done in a variety of different ways. However, as the matrix exponential is multiplied with the vector x_0, we do not need to compute the matrix exponential as a full matrix, but only the action of the matrix exponential on the vector x_0. Algorithms for this task are studied in the context of exponential integrators [7, 8].

The definition of the matrix exponential directly yields that the matrix exponential of a triangular matrix (as it is in our case) is again a triangular matrix.

The matrix A in the linear dynamical system will be the Kronecker sum of several matrices (Eq (13) in the main text). The matrix exponential of such a matrix simplifies according to [9, Theorem 10.9, p. 237] to

$$\operatorname{expm}\left(\bigoplus_{i\in[n]} A_i \right) = \bigotimes_{i\in[n]} \operatorname{expm}(A_i), \tag{S1-10}$$

where $[n]$ denotes the set of integers from 1 to n.

Thus, instead of computing the matrix exponential of one large matrix, we can compute the matrix exponentials of several small matrices and connect them with the Kronecker product, which gives an additional performance boost for numerical computations.

The Eq (S1-10) can be seen as a generalization of $e^{a+b} = e^a e^b$ for real numbers a and b. However, it is important to note that this statement does not hold for the standard matrix addition and product. Thus, in general, we have [9, Theorem 10.2, p. 235]

$$\operatorname{expm}(A + B) \neq \operatorname{expm}(A) \operatorname{expm}(B). \tag{S1-11}$$

In the model components for dependent mutational processes, we consider sums of matrices (see Eq (10) in the main text).

While in this case the matrices cannot be simplified as much as for the independent model component, we can still use the exponential integration mentioned above. Further mathematical analysis is however possible in the context of operator splitting [10] or the Baker-Campbell-Hausdorff formula [11].

Often, the initial value x_0 itself can be written as a Kronecker product, i.e.,

$$x_0 = \bigotimes_{i \in [n]} x_i, \tag{S1-12}$$

where the x_i are vectors with sizes corresponding to the sizes of the matrices A_i. With this, the solution of the dynamical system (S1-7) can be expressed as

$$x(t) = \left(\bigotimes_{i \in [n]} \operatorname{expm}(tA_i) \right) \left(\bigotimes_{i \in [n]} x_i \right) = \bigotimes_{i \in [n]} \operatorname{expm}(tA_i)\, x_i, \tag{S1-13}$$

where the last equality is due to the mixed product property of Kronecker products [3].

In most cases, we are not only interested in a single entry of the solution vector $x(t)$, but in the sum of several entries. To achieve this, we consider the scalar product $v^\top x(t)$ of the vector $x(t)$ with a vector v of the same size which has a 1 in all states we want to consider and a 0 everywhere else.

Often this vector can, similarly to the initial value x_0 in Eq (S1-12), be written as the Kronecker product of vectors v_i with sizes corresponding to the matrices A_i

$$v = \bigotimes_{i \in [n]} v_i. \tag{S1-14}$$

In this case, the accumulation simplifies to

$$v^\top x(t) = \bigotimes_{i \in [n]} v_i^\top \operatorname{expm}(tA_i)\, x_i = \prod_{i \in [n]} v_i^\top \operatorname{expm}(tA_i)\, x_i, \tag{S1-15}$$

where the first equality follows as above from the mixed product property of Kronecker products and the second one is due to the fact that the Kronecker product of real numbers (here: $v_i^\top \operatorname{expm}(tA_i)\, x_i$) is the standard product of real numbers.

5 Introducing a Death State

The introduction of a state for dead or disappearing crypts is natural from a mathematical point of view. We collect the probabilities that a crypt with a certain genotype dies in a vector d, which has as many entries as there are genotypes.

The state vector of the linear ordinary differential equation (10) in the main text is extended by an additional state x_{d} which models the number of crypts in the death state, i.e., crypts that have disappeared. Similarly, the matrix is extended by one row and one column to include the probability vector d. This leads to the linear ordinary differential equation

$$\begin{pmatrix} \dot{x}(t) \\ \dot{x}_{\mathrm{d}}(t) \end{pmatrix} = \begin{pmatrix} A & d \\ 0 & 1 \end{pmatrix}^{\top} \begin{pmatrix} x(t) \\ x_{\mathrm{d}}(t) \end{pmatrix}, \qquad x(0) = x_0, \quad x_{\mathrm{d}}(0) = 0. \tag{S1-16}$$

The 0 in the matrix denotes a row vector containing only zeros. Its interpretation is, that a dead crypt cannot move to a different state, i.e., become alive again, but stays dead, which is also expressed by the 1 in the matrix. The initial condition $x_{\mathrm{d}}(0) = 0$ means, that there are no death crypts at time $t = 0$.

As above, we write the solution of this linear ordinary differential equation using the matrix exponential

$$\begin{aligned} \begin{pmatrix} x(t) \\ x_{\mathrm{d}}(t) \end{pmatrix} &= \operatorname{expm}\left(\begin{pmatrix} tA^{\top} & 0 \\ td^{\top} & t \end{pmatrix} \right) \begin{pmatrix} x_0 \\ 0 \end{pmatrix} \\ &= \begin{pmatrix} \operatorname{expm}\left(tA^{\top}\right) & 0 \\ td^{\top}\varphi\left(tA^{\top}\right) & \exp(t) \end{pmatrix}^{\top} \begin{pmatrix} x_0 \\ 0 \end{pmatrix} \\ &= \begin{pmatrix} \operatorname{expm}\left(tA^{\top}\right) x_0 \\ t\ d^{\top}\varphi\left(tA^{\top}\right) x_0 \end{pmatrix} \end{aligned}$$

Hereby, φ denotes the function $\varphi(x) = \frac{e^x - 1}{x}$, which is extended to matrix arguments in the standard way [9]. The second equality follows from (a slightly modified version of) Theorem 1 in [12].

This means that — when using the same parameters as in the original model — the

number of crypts of a certain genotype is not changed compared to the original model. The number of crypts in the death state is given by $x_{\mathrm{d}}(t) = t\ d^{\top} \varphi\left(tA^{\top}\right) x_0$.

References

1. Hammack R, Imrich W, Klavžar S. Handbook of Product Graphs. 0th ed. CRC Press; 2011.

2. Horn RA, Johnson CR. Topics in Matrix Analysis. Cambridge: Cambridge University Press; 1991.

3. Loan CFV. The Ubiquitous Kronecker Product. Journal of Computational and Applied Mathematics. 2000;123(1-2):85–100. doi:10.1016/S0377-0427(00)00393-9.

4. Kaveh A, Rahami H. A Unified Method for Eigendecomposition of Graph Products. Communications in Numerical Methods in Engineering. 2005;21(7):377–388. doi:10.1002/cnm.753.

5. Teschl G. Ordinary Differential Equations and Dynamical Systems. Providence, R.I: American Mathematical Society; 2012. Available from: `https://bookstore.ams.org/gsm-140`.

6. Moler C, Van Loan C. Nineteen Dubious Ways to Compute the Exponential of a Matrix, Twenty-Five Years Later. SIAM Review. 2003;45(1):3–49. doi:10.1137/S00361445024180.

7. Al-Mohy AH, Higham NJ. Computing the Action of the Matrix Exponential, with an Application to Exponential Integrators. SIAM Journal on Scientific Computing. 2011;33(2):488–511. doi:10.1137/100788860.

8. Niesen J, Wright WM. Algorithm 919: A Krylov Subspace Algorithm for Evaluating the φ-Functions Appearing in Exponential Integrators. ACM Transactions on Mathematical Software. 2012;38(3):19. doi:10.1145/2168773.2168781.

9. Higham NJ. Functions of Matrices: Theory and Computation. Society for Industrial and Applied Mathematics; 2008.

10. McLachlan RI, Quispel GRW. Splitting Methods. Acta Numerica. 2002;11:341–434. doi:10.1017/S0962492902000053.

11. Biagi S, Bonfiglioli A. An Introduction to the Geometrical Analysis of Vector Fields: With Applications to Maximum Principles and Lie Groups. WORLD SCIENTIFIC; 2018.

12. Sidje RB. Expokit: A Software Package for Computing Matrix Exponentials. ACM Transactions on Mathematical Software. 1998;24(1):130–156. doi:10.1145/285861.285868.
