# Supplementary material for: Mathematical modeling of multiple pathways in colorectal carcinogenesis using dynamical systems with Kronecker structure
Source: PLoS Comput Biol. 2021 May 18;17(5):e1008970. doi: 10.1371/journal.pcbi.1008970 (PMC8162698; doi:10.1371/journal.pcbi.1008970)
Supplement: Attachment — Submitted filename: Point-by-point_response.pdf [file pcbi.1008970.s002.pdf]

Point-by-point response to reviewers' comments

We thank the reviewers for their thoughtful comments and suggestions. As some of the reviewers' comments were referring to medical assumptions underlying the model and the parameter setting, we would like to comment on this beforehand to clarify these important points and shorten the following responses.

In general, a sequence of mutations can cause cancer. As different driver mutations occur in varying order, there are multiple molecular pathways of carcinogenesis with impact on the course of cancer and on the preventability of precursor lesions. We provide a general mathematical framework that describes arbitrarily complex and arbitrary numbers of pathways and mutations because the chosen Kronecker structure enables a modular construction and an analytic, computationally efficient solution.
We use Lynch syndrome (LS) carcinogenesis to illustrate the flexibility of the model. Naturally, specific assumptions may vary for other types of cancer. We illustrated model modifications for FAP, Lynch-like and the classical colorectal carcinogenesis.

There are two types of **assumptions** in the approach: *1.* Assumptions underlying the mathematical model and leading to the special Kronecker structure, and *2.* Assumptions for the precise parameter setting in the example application of LS-associated cancer. Whereas the former are present in all model applications, the latter are application-specific and can be adapted to the data at hand. We shortly comment on these two points:

1. The mathematical model consists of components for independent (proven independence or no evidence of dependence) and dependent mutation processes. **The independence component** is based on three assumptions: 1) All combinations of mutations in the considered genes are possible without any additional genotypic states, 2) no simultaneous mutations occur, 3) mutational processes are independent. Assumption 1 is necessary only for the underlying mathematical structure, but not a limitation from the medical point of view. The assumptions 2 and 3 allow for modeling independent mutations. As also mentioned by the reviewers, independent mutations are not always biologically correct. Thus, **the dependence components** explicitly include dependent and simultaneous mutations. For the example of LS, we present adaptations for increased point mutation rates (*APC* and *KRAS* after MMR deficiency, matrix *B* and *F*), simultaneous mutations (*CTNNB1* and *MLH1* alterations, matrix *C*), an increased LOH rate (after *APC* inactivation, matrix *D*). Some effects become mutually enhancing, which is implemented by an additional matrix (matrix *E)*.
2. To obtain exemplary results in the LS scenario, we choose the assumptions of Section 3.2 and 3.3 for filling the definitions of gene-dependent point mutation and LOH rates with

numbers. As also pointed out by Reviewer #2, the general approach of defining mutation rates in a gene-dependent way, was also proposed in Paterson et al, PNAS, 2020. How the chosen parameters influence the results is already discussed in the manuscript in Section 5.4 “Analysis of Parameter Contributions”. However, it should be noted that the assumptions for the specific parameters only affect the specific example to illustrate the performance of the model.

To address the reviewers’ concerns and to clarify the modeling approach, we have restructured the modeling section with one section each for independent and dependent mutational processes. We sharpened the Abstract and Introduction (Reviewer #3), added a discussion of the parameters to the Contribution and Discussion sections (Reviewers #1, #2). We replaced Fig. 4 (matrix structure of the basic component) with the structure of the system matrix built additively by the different model components (now Fig. 3). The same applies for the Graphical Abstract c.

Reviewer #1:

I below comment on the starting points/assumptions for the reasoning and leave the correctness of the specific mathematics and its references to a professional statistical expert. The paper is very difficult to read, partly because lack of clear organization: Neither the definition of terms used, the basic conceptional understanding of what is ment by ‘cancer’ (and which seemingly is confused with ‘tumor’ in parts of the mns) and the basic assumptions underlying the statistics are not presented distinctly do the reader, and the obligatory section discussing the impact of these/violation of these I do not find.

Thank you for your comments. Besides the general answer above, definitions of key terms have been harmonized in the manuscript. Regarding the reviewer’s last point: We restructured Section 6, now titled Discussion, and discuss the implications and limitations of the assumptions of the model (see second following comment).

Deep inside the text I find three key factors mentioned:

1. *the order in which mutations are accumulated is ignored*
2. *The edge connectivity assumption states that no two mutations in different genes can occur at the exactly same point in time*
3. *The third assumption entails that a mutation in one gene does not change the mutation probability in another gene.*

the first of which seemingly in conflict with the basic understanding that the order of the events is of interest as mentioned initially, the second partly invalid, and the third obviously

wrong: If an initial mutation increases proliferation rate, the probability for one of the cells carrying the mutation to have a second hit is to me obviously increased – which is what we teach the student when explaining carcinogenesis. And which is why the order of mutations is believed to be significant. I do see that there may be ways to read the paper not to find these arguments to be contradictory, but a scientific paper is not to be text to be read in good faith to be understood correctly.

We are grateful for the comment. We have profoundly re-structured the sections describing the assumptions and their implications (see general comment above). The assumptions referred to by the reviewer represent base assumptions for independent events. They are adapted to the biological reality in the dependency section (see pp. 18).

The shifts back-and-to between considering cancer/tumor in general and MMR-deficient tumours specifically has to me the flavor of a paper explaining something to the general public with examples, and is not what is expected in a scientific paper. These shifts make it very difficult to control the relationships between assumptions/input to calculations and results.

We thank the reviewer for this input. Following Reviewer #3, we restructured the first sections (Introduction, Medical Background, Related Work, Contribution) and revised the terminology. Mathematical Background can now be found in the Appendix. The modeling approach is now presented in Section 3, including the different model components for independent and dependent mutational processes. Section 4 introduces modifications of the model to account for other types of cancer (FAP, Lynch-like, MSS) while Section 5 presents the simulation results. Section 6, now named Discussion, is completely restructured, discussing the assumptions and their implications.

The intro mention immune system counteracting cancer development, but does not refer to the growing knowledge of 'overdiagnosis' – established cancer may stop growing and/or disappear. By and large the mns seemingly consider cancer a result of a process having some probability to occur, but does not consider the common understanding that these biological events are much more frequent than the end result cancer indicates because there are opposing system removing the mutated cells – it's briefly mentioned but not discussed to my reading as a major factor. When considering Lynch Syndrome, this is of importance because the initiating factor (MMR deficiency) may/will all along the way constantly impose new mutations which may both block the effect of first mutations and trigger new biological systems with the effect of Darwinistic reduced fitness of the growing cells/clone. This to me is an element of probability, and which seemingly is denied in the assumptions: "Most of the

cancers in the general population occur by chance. These cancers are called sporadic. However, in some families, certain types of cancer appear more frequently. This is either a familial or a hereditary form of cancer…. From a modeling point of view, the advantage of focusing on hereditary tumors is that there are clearly defined molecular events determining the onset of the disease and thereby representing a known mechanism underlying carcinogenesis." To this referee, these assumptions are questionable, I would rather say that any individual has a large set of inherent and acquired probabilities for biological mechanisms going wrong, and the difference between familial/inherited cancers and 'sporadic' ones is that the familial/inhered cancers cases have different probabilities for some unwanted outcomes in the billions of biological events happening in their cells each second they live. Following such a way of thinking, cancer is close to inevitable if one live long enough, but with increasingly good health and longliving the probability for contracting cancer increases because the incidence of dying before you so do decreases. In such a context, the probability for being live born and live for many years with a significant failure in one of the important system to mend errors, is about a small brim of errors between what is 'normal' and what is compatible with life. Boiling it down to a short analogy: The human body is not a machine, because it has the inherent capability of repairing itself, and to me this basic understanding is not good enough visible in the mns. In sum, to me the mns was interesting reading, but the validity of the conclusions I cannot describe because the authors to me don't.

Thank you for highlighting the very important point of overdiagnosis and disappearing lesions, which we added in the Medical Background section. However, there are currently not enough data available to estimate or learn the necessary parameters, i.e., for each mutation status the probability that a crypt dies. This is the reason, why we have chosen a somehow simpler model where the proliferation and disappearing of crypts with certain genotypic states is jointly modeled by the self-loops in the graph, largely reducing the number of probability parameters we need to determine. We added a modeling idea in Section 3.4 and in the Appendix A.5, highlighting the model's flexibility to address disappearing lesions in the Discussion section.

Reviewer #2:

This is a solid paper that describes a mathematical approach to deterministic modeling of cancer initiation. I have two concerns about the paper: 1) their model is extremely similar to the recently published Paterson et al. paper (Ref. 37) and 2) many of the parameter values are arbitrarily set, even when estimates are available from the literature.

Regarding issue 1), the authors mention the Paterson et al. paper, but are not clear as to how much of their model is virtually a verbatim copy of the Paterson et al. model. They

consider 5 genes, APC, KRAS, TP53, CTNNB1 and "MMR", whereas Paterson et al. consider APC, KRAS and TP53. The graph of genotypes and the possible states for the three genes in common are exactly the same as in Paterson et al, as well as the general approach to calculating gene-specific numbers of driver positions, studying accumulation of mutations in a population of crypts (i.e. disregarding tissue hierarchy), assuming APC and KRAS provide fitness advantage but not TP53 etc. The Paterson et al. model is stochastic, but the authors use a dynamical system approximation to derive analytic results, which is also similar to the present paper. Even though the focus of the manuscript is on Lynch syndrome, the authors should clearly state that the approach they take is very similar to the one of Paterson et al. in many aspects.

Thank you for highlighting this. We fully acknowledge certain similarities to the model published by Paterson et al., which we have already referred to in the manuscript. However, our model is fundamentally different from the model by Paterson et al. and extends the applicability for modeling biological processes in important aspects:

Linear ODE vs. Stochastic processes: The model by Paterson et al. uses a linear ordinary differential equation (ODE) for all genotypic states except for the transition to the last (cancerous) genotypic state. This last transition is modeled using a stochastic branching process. Thus, the Paterson approach is a hybrid approach of linear ODEs and a branching process. In contrast, we use a system of ODEs to model the evolution of all genotypic states which simplifies the computational solution process tremendously. This adds versatility and applicability to different scenarios of carcinogenesis in a straightforward way.

Dependent mutational processes: While the Paterson et al. model is based on independent mutation rates, our model consists of different components for modeling independent and dependent mutational processes. As pointed out by Reviewer #1, the assumption of independent mutational processes only reflects biological processes to a limited extent.

Number of cancerous genotypic states: Paterson et al. consider only one cancerous genotypic state. However, our model incorporates the possibility for multiple cancerous genotypic states reflecting the real-world heterogeneity of cancer. This is important because differences in cancer phenotypes could be accompanied by measurable clinical differences and therefore should be reflected in a modeling approach.

Different initial values: Paterson et al. approach considers as initial value only wild-type crypts. Modifying the initial value to model Lynch syndrome or FAP would completely change the derived approximations. By choosing different initial values, our model is capable of accounting for different scenarios, including hereditary cancer predisposition syndromes.

Extensibility to more genes: The Paterson et al. approach is based on three defined genes *APC*, *KRAS*, *TP53*. Including more genes would heavily enlarge the definition of the ODE in

the Paterson model. Because of the Kronecker structure, our model can be extended to more genes in a straightforward way.

Approximations vs. exact computations: The approach by Paterson et al. makes several simplifying assumptions to derive approximations. Contrary to this, our model does not use any approximations. All formulas are exact from a mathematical point of view.

We have added a paragraph explicitly addressing these aspects.

My second issue is regarding how parameters were set. For example, regarding results in Fig 10 the authors state: "Number of MMR-deficient Crypts over the Life of a typical Lynch Syndrome Patient for MLH1 and MSH2. The parameters in the model are set in such a way that the simulation results are in concordance with published data". Why did the authors choose to set parameter values in this way? Since there are many parameters in the model, how did they choose these specific values? A better approach would have been to estimate all parameters from the literature, whenever possible. For example, b(KRAS) has been estimated to be 0.07/year (Ref. 67), and the authors set it to 0.01. Estimates similarly exist for b(APC) (Ref. 13).

Thank you for this question. As outlined above, the chosen parameters only affect the specific example and do not have any impact on the general model (see above).

For the example of Lynch syndrome, no precise estimates on b(KRAS) and b(APC) exist. The exact parameter values for the self-loops in Paterson et al, PNAS, 2020 resulted in simulations with ~10^8 APC-/- crypts by age 80 years (Fig. 3A), meaning that all crypts in the colon of all patients are APC inactivated, which does not seem plausible. In Section 5.4 of our manuscript, we analyze the influence of different parameters on our simulation results, underlining the fact that the exact parameter values used in the example only represent one possible setting in a reasonable range to illustrate the general modeling approach.

We did not include the procedures for parameter learning and sensitivity analysis as this would have unduly extended the manuscript. We are planning a more mathematically focused follow-up work that explains the parameter learning in more detail. A respective paragraph was added to the manuscript.

There are also some formatting issues, e.g. eq. (1).

We hope that there were no errors in the assembled manuscript version available to the reviewer due to conversion of formulas. The small square symbol □ is the mathematical symbol for the Cartesian product of two graphs. We added an explanation accordingly.

Reviewer #3:

The manuscript entitled "Mathematical Modeling of Multiple Pathways in Colorectal Carcinogenesis using Dynamical Systems with Kronecker Structure" by Haupt and colleagues propose an extensible mathematical model of colorectal carcinogenesis for Lynch syndrome based on Kronecker sum of mutation graphs adjacency matrices. The authors focus on an important problem, however they hardly put their work into context. Modeling hereditary colorectal cancer in terms of genomic alterations occurring in defined pathways is interesting, however the enthusiasm for this work is highly diminished by a few major shortcomings, as discussed below.

Major comments:

1) it is not clear what purpose this whole exercise will serve. What does this model does for a patient? The authors mention vaguely "individual needs for prevention and treatment", but they do not make any effort to develop this point.

In the current form, the model is not for individual patient predictions but for analyzing the general relative contributions of the molecular pathways of carcinogenesis among all cancers of this type. Further, cancer risk estimations in subgroups of carriers can be made and the effects of prevention measures affecting single pathways on the overall contribution of the pathways can be analyzed. In Lynch syndrome, this concerns the possible influence of MMR gene germline variants on cancer incidence and precursor lesions (Fig. 9) and the effectiveness of tumor preventive measures, like colonoscopy or vaccines, targeting specific pathways of carcinogenesis.

2) The mathematical modeling is very complicated, for sure inaccessible to the (lay) audience working in the cancer research field. The authors should make a substantial effort to make it less obscure.

We tried to thoroughly revise our manuscript, leaving the mathematical details as appendix for the readers interested in the mathematical background of the presented model. In addition, we have restructured the modeling section now including different parts for the model components (see also the general answer above). We added paragraphs in the introduction explaining the model structure to the medical community. The mathematical details are targeted at Mathematical Oncologists.

3) The mathematical framework appears to be "ad hoc", with many assumptions that are not necessarily adequate, justifiable, or justified, so it is hard to appraise how generalizable this approach would be. Even the choice to focus on Lynch syndrome is partial, since this is not the "mainstream" path to carcinogenesis.

By the general answer above, our approach is indeed generalizable to different types of cancer, as also explained and shown in Sections 4 and 5.5. This is possible in a straightforward manner by including different genes and genetic dependencies. Lynch syndrome carcinogenesis serves as a valuable example since it shows the nature of multiple molecular pathways of carcinogenesis.

4) The paper is, in summary, a very long list of assumptions. Some of them questionable from the cancer biology point of view. Some sound, some very well explained, some not explained that well. An effort should be made justify all assumptions. That said, how generalizable is this approach if it depends on so many assumptions?

Thank you for your questions. The model itself is based on very few general assumptions, with the explicit aim to assure general applicability of the model. Additional biological assumptions are for illustration purposes only and do not affect generalizability (see general answer).

Below follows a few comments to support my review.
Specific comments:
1) Abstract:
- The abstract requires major revision: after reading one can only guess what the manuscript is about. It appears it is about tumor evolution in a very specific hereditary syndrome, and that this should be important for developing more rational clinical treatments. Yet, this is not put into context, and the discussion of the mathematical models and their assumptions remains vaguely linked to the problem under investigation.

We thank for the reviewer's input. We revised the abstract which is now given in a structured form. We included an explanation of the different molecular pathways in colorectal carcinogenesis (see also comment below). Further, we added medical context by including a detailed description of the simulation results for Lynch syndrome carcinogenesis.

- The authors, in the abstract and introduction, make claims about "different types of colorectal cancer with individual needs for prevention and treatment." Yet these are not introduced and discussed. Explicitly grounding the work in current colorectal cancer research (and the related knowledge on molecular subtypes, etc...) would be highly beneficial.

In general, the subclassification of cancer based on molecular parameters becomes increasingly important in the last years, as shown for example for colorectal cancers in Guinney et al, Nature Medicine, 2015 which we added to the manuscript.
For the presented example of Lynch syndrome, recent research has suggested three

molecular pathways of colorectal carcinogenesis (Ahadova et al., IJC, 2018): One starting with adenoma formation, then MMR deficiency and tumor outgrowth; the second initiated by MMR deficiency, then adenoma formation and tumor outgrowth; and the third MMR deficiency as initiating event and invasive tumor growth. Those are linked to different mutational processes and clinical course. We added these points in the Medical Background, Section Lynch syndrome-associated colorectal carcinogenesis.

- I definitely agree with the idea that "... a single model describing the whole process of carcinogenesis ... is too complex to build". I think that this issue is grounded in the reductionist (and often positivist) approach of most scientific research in this fiels (and others too, of course). And yes, "... the increasing medical knowledge ..." had made it more evident. Yet, even postulating the possibility (of a unifying theory) in the first place might be perhaps too ambitious. That said, the authors should perhaps consider to restructure the Introduction section to make it more consequential. Discussing mathematical modeling before discussing cancer and its pathogenesis is a little odd, and does not help the readerships to follow the narrative. More explicitly: sections 1.1 and 1.2, should go after Section 2, and maybe section 1.3 should come at the beginning.

Thank you for your input. We changed the order of the sections and rephrased accordingly.

- Citation 16 might not be the correct one: did the authors meant to cite: "doi: 10.1093/bioinformatics/btp505"?

We fully agree with the reviewer. In the first version of the manuscript, we stated the paper with the mathematical foundations. The given citation directly refers to the medical application. We have changed the reference as suggested.

- Lines 109-112: "..., while loss of heterozygosity (LOH) refers to the loss of larger regions of a chromosome, which can result in the deletion of whole genes." -> This is not correct, or well explained. LOH is not COPY NUMBER LOSS or COPY NUMBER ALTERATION... LOH is when one copy of a normally diploid genome is lost at some resolution (a focal loss, or a larger region, or a whole chromosome, it doesn't matter). This can result in loss of function, when coupled with mutations (or other alteration types) in the remaining copy. Please correct.

Thank you for clarifying. We corrected the explanation accordingly.

- Lines 113-119: Discarding 99% of the genome is "old school". Pathogenic, non-coding mutations affecting coding genes have been known for years (see the globin genes in

thalassemia for instance) and the relevance of non coding genes is becoming more and more relevant. Hence, the following statement should be amended: "As about 99 % of the genome is not translated into proteins, most mutations do not have a direct consequence on cell viability or behavior." - Lines 120-121: See above, therefore the following statement – "This means that, from all the possible mutations that can occur, we need to identify the relatively few key events which have a functional impact on the cell" – is not entirely correct and should be revised. There might be only a few CODING mutations that might be relevant, however we have no clues about the non-coding domain.

We deleted the first sentence and adapted the second one.

- Lines 123-125: There is also the possibility that a cancer is controlled by non-cell autonomous mechanisms, that are not defined by the combination of mutational events happening in the initiating cell (i.e., affecting oncogenes, tumor suppressors, and the detrimental mutations affecting the fitness of the cancer "to be" cell)... For instance, the role of immune surveillance... etc.

We fully agree that a holistic view of cancer development has to consider non-cell autonomous mechanisms like immune surveillance. Our model, through the flexibility regarding mutational events and their consequences, can also be used to implement external influence factors, e.g., tumor-immune cell interactions, for example assuming a higher immune visibility of MMR-deficient cell clones with high mutation load. We are currently working on an expansion of the present model in a tissue context. Corresponding sentences have been added to the Medical Background and Discussion section.

- Lines 126-128: "Different combinations of key mutations result in several distinct pathways." this sentence does not make sense. Signaling pathways exist independently from the mutations in the genes that encode the proteins participating in said pathways. Different combinations of mutations result in aberrations of specific pathways, usually non redundant ones. Furthermore, pathways don't "arise", they exist in a normal state, they dynamically regulates various processes in cell and tissue homeostasis, and are altered in response to genomic hits (whatever their nature is).

We are sorry about the confusion with the word “pathway”. In our manuscript, we use the term referring to pathways of carcinogenesis, according to common use in the field (Ahadova et al., IJC, 2016 and Tan, Yan, Biology Direct, 2010). We added respective explanations and changed the term to “pathway of carcinogenesis” where applicable.

- Lines 128-131: The authors should put this "high-dimensional and complex problem" into context. Perhaps they should mention that a tumor harbors hundreds/thousands of mutations, and that they are scattered over many genes/pathways/processes, etc... Perhaps they should also define the "problem". Is the the problem making sense of all the possible mutation combinations, finding the one or few combinations of drivers? If yes, for a given cancer? For all cancers in a population? Since it is also mentioned "medical interpretability", are the authors referring to individual patients? If so, it should be explicitly stated and explained.

Thank you for your questions. When restructuring the introduction, we moved this part to the end of Paragraph “Lynch syndrome-associated colorectal carcinogenesis” and explained these points in more detail. The exemplary problem to be addressed by our model is to analyze the relative distribution of molecular pathways of carcinogenesis among all Lynch syndrome-associated cancers and in particular not about individual patient outcomes. For the medical interpretability, we refer to our answer to your first question.

- Lines 134-136: "Mutations are errors which occur during DNA replication within cell division and which are not repaired by one of the error detection, repair and control systems present in all organisms." It is odd to explain the biochemistry of mutations in details here, after having discussed them before in the manuscript, giving for granted the reader was already well acquainted with the concept.

Thank you, we removed this part, and restructured and rephrased the respective paragraph.

- Lines 147-152: perhaps when distinguishing hereditary from sporadic cancers the authors could introduce the concept of somatic versus germ-line mutation. The explanation provided is confusing, for instance using the word "genetic" ("The former is due to a combination of genetic and environmental factors...") points to the genetic material, the DNA, but it might also suggest heritability, etc... And so on... Making the distinction between "somatic" and "germ-line" mutation is key when discussing sporadic and hereditary cancers... Especially since somatic mutations are mentioned later on in the paragraph (line 158).

We added an explanation of somatic mutations and inherited germline variants to make this point clear (see also Comments by Reviewer #1).

- Lines 168-173: Here it should perhaps be discussed the existence, in the crypts, of stem cells, and then mention tissue renewal, and why it is important to look at stem cells and hence crypts…

Thanks for mentioning this essential point. Modeling monoclonal conversion and analyzing the role of colonic stem cells located at the crypt base is a major task for future approaches to understand the intra-crypt dynamics. We are currently working on a computational model with first results available as a preprint (Haupt et al, bioRxiv 2020.12.29.424555, 2020) and added a paragraph discussing the importance of intra-crypt dynamics for carcinogenesis.

- Lines 180-185: here the authors seem to imply that MMR genes are not to be considered tumor suppressors... If they are not tumor suppressors, what are they? Aren't tumor suppressor genes involved, broadly, in these following processes? 1) Stop cells from dividing; 2) Repair DNA damage; and 3) Start programmed cell death. So, it appears that MMR are bona-fide tumor suppressors…

There have been discussions about the terminology of bona fide tumor suppressor genes in the past. The distinction between growth-regulatory and DNA repair type tumor suppressor gene has been discussed extensively in the scientific literature. As the terminology has no impact on our modeling approach and the results, we decided to avoid ambiguous terms and shortened this paragraph following the reviewer's suggestion.

B) Introduction – Section 3.
- Lines 215-217: "... the accumulation of mutations in two different genes of the same cell, ...", perhaps the authors want to say the "OCCURRENCE of mutations", since rarely in the same cell mutations accumulate in the same gene…

We fully agree and corrected the term where appropriate. According to your suggestions and a comment of Reviewer #1, we put the Section Mathematical Background in the Appendix.

- Lines 230-237: about the "Independence of the Processes" it is stated that it "... entails that a mutation in one gene does not change the mutation probability in another gene." Is this assumption reasonable? MMR mutations are exactly the mutations that makes other mutations more likely. Similarly, mutations in other tumor suppressor genes are linked to subsequent genomic alterations, therefore this assumption is not correct. The possibility that this could be useful to create a "baseline" to measure how much this assumption is violated is besides the point... What value there is in measuring the deviation from a "wrong" baseline? The model derived can still be god at describing tumorigenesis. At least the authors could make the argument that this wrong assumption is necessary to start working, then... maybe down the road they will be able to revisit the whole thing and make better assumptions…

Thank you for highlighting this very important point (see also general answer above). We fully agree that there are dependencies between mutations. Although the Cancer Dependency Map (www.depmap.org) provides a great resource and extensive information about gene dependencies, data for specific medical contexts are scarce. To allow implementation of new gene dependency information, our model baseline is neutral and starts with the artificial assumption of complete independence. The process of adaptation to known dependencies is illustrated in our example of Lynch syndrome carcinogenesis.

C) Section 4.1

- Lines 313-316: When the authors discuss the "ll" state, they mention that "... in CTNNB1, APC and TP53 damage a cell in such a way that it directly leads to cell death. Thus, there will be no crypt with all cells being in that state. As we model the evolution of genotypic states of crypts, we do not consider the ll states for CTNNB1, APC and TP53." It is not clear whether this assumption is reasonable. Let's take into consideration TP53. Usually mutations in this genes are dominant negative, so one hit is enough to have a phenotype, so to speak (despite TP53 being considered a tumor suppressor). So, parsimony in tumor evolution might explain better the absence of the "ll" state for this gene, rather then lethality... Furthermore, it is not clear why to distinguish "ll" from "mm"... Also two hits of "m" types can potentially be lethal, so it is not clear why to make a special case of "ll" and not "mm", or "ml" for these genes... This assumption might be justifiable, in some ways, even it could be grounded in tumor biology, but the authors should make a better case for it than they do. They could look into large genomic studies, and for instance have a look at the frequencies of double hits in their favourite genes, and perhaps empirically justify their assumption... It is true that TP53 is very rarely mutated twice, etc…

In this regard, we used the same assumption as the model by Paterson et al, PNAS, 2020. Quoting from this paper (see section Results, Model and Parameters), "Loss of both alleles is not allowed, as this is usually fatal for the cell." We added the citation at the respective sentence in our manuscript. However, if this assumption, in the future, turns out to not be reasonable, our model can be adjusted quite easily by adding the "ll" states for those genes in the gene mutation graphs. This increases the system dimensions but leaves the model structure unchanged. We added a sentence to the Limitations section in the conclusion.

- Lines 319-322: these lines makes the assumption above even more confusing: the authors knows that TP53 is consider a tumor suppressor, they also know about dominant negative effect, yet... While they ignore this effect for APC, they make no comment about TP53... In other words, without some clear consequential explanations, it might look like the authors "bend" cancer biology as it is necessary.

We completely share the reviewer's view about the significance of dominant negative TP53 mutations. We adapted the statement in the manuscript explaining that the exemplary application of the model ignores dominant-negative mutations in concordance with Paterson et al, PNAS, 2020. As outlined above, including these effects is possible in the future.

Additional minor comments:

1) Abstract: "Like many other tumors, colorectal cancers develop through multiple pathways and different driver mutations." -> I would rephrased this stating that "... colorectal cancers through ALTERATIONS AFFECTING multiple pathways RESULTING FROM different driver mutations".

We are grateful for the comment. The term "pathway" has been clearly defined as "pathway of carcinogenesis" in the revised version of the manuscript as described above.

2) Abstract: "e suggest a linear autonomous dynamical system modeling the evolution of the different pathways of colorectal carcinogenesis." -> Unclear, please rephrase.

According to the previous comment, this should now be clarified.

3) Figure one would be better explained by labeling the panels and then explaining them accordingly in the corresponding legend.

Thank you. We adapted the figure and reference them now accordingly in the manuscript.

4) Introduction: references for the Lynch syndrome, MSI, etc should be added.

We added references for Lynch syndrome and MSI in general (Boland and Goel, Gastroenterology, 2010; Jasperson et al, Gastroenterology, 2010; Kloor and von Knebel Doeberitz, Trends in Cancer, 2016) and for current cancer research in Lynch syndrome pointing to the need of adapted prevention strategies (Engel et al, Gastroenterology, 2018; Ahadova et al, IJC, 2020; Engel et al, Gastroenterology, 2020) also mentioning disappearing lesions due to immune surveillance and overdiagnosis (see also Reviewer #1, Seppälä et al, Hereditary Cancer in Clinical Practice, 2019).

5) Please check format of references 9 and 10.

We checked the format and corrected spacing.
