# Supplementary material for: Mathematical modeling of multiple pathways in colorectal carcinogenesis using dynamical systems with Kronecker structure
Source: PLoS Comput Biol. 2021 May 18;17(5):e1008970. doi: 10.1371/journal.pcbi.1008970 (PMC8162698; doi:10.1371/journal.pcbi.1008970)
Supplement: Attachment — Submitted filename: Point-by-point_response.pdf [file pcbi.1008970.s003.pdf]

Point-by-point response to reviewers' comments

Reviewer #1:

Firstly, I am grateful for the opportunity to discuss this paper with the authors, and trust them to understand that my expected contribution is to discuss problems and neither to give global statements nor promote parts to which I may agree. I also take the opportunity to add that the problems the authors address are well known to me, as are the difficulties to describe them in mathematical models which are as simple as possible (Descartes) but not too simple (Einstein). When IT systems now have the ability to undertake the numeric complexity of the analyses described, time is due to do so. When no one has all the knowledge needed to do so alone, each of us will have different focus. Mine are the assumptions used in the mathematical algorithms and their consequences.

In general, the authors have responded to my initial comments, but the mns is still very complex with many words and I still find problems needed to be clarified. I discuss these below partly in the order spotted when reading the revised mns and partly trying to make a readable narrative, not in the order of importance as is usual – because it is unclear to me to which degree they may modify the results, and the identification of the problems may be the only step to currently agree upon, and which in itself is the requirement to at some time to arrive at a conclusion.

If I had a solution to the problems I describe below, I would have submitted a paper with these myself, but I have not. In part the solution may be there is no solution, to which I may refer interested to the conflict between the works/conceptions by Newton and Hawkins on causation and probabilities (to put it simply: a rare stochastic event does not have a cause). My intention with describing the problems below, is to ask for a more clear definition of the terms used, the assumptions used, and a more distinct discussion of the limitations of the conclusions arrived at.

We are grateful to the reviewer for the thorough considerations and happy to discuss the natural limitations of mathematical models based on current knowledge. We fully agree that cancer evolution is a highly complex process, and solving the unsolved problems requires scientific exchange between different disciplines. In this regard, the reviewer addresses many key aspects that we tried to incorporate into the revised version of our manuscript.

The definition of 'cancer' is still lacking, and combined with the terminology 'a linear model' and 'cause of death' one get the understanding that an untreated cancer will cause death. In contrast, the many crypts in the gut are constantly proliferating and the cells are expelled into the lumen and die. This may be the fate of MMR deficient crypt cells – growing precancers but not visible tumours because they are – in contrast to adenomas - constantly loosing their mass. Also, the immune system is known to recognize MMR deficient cells as 'foreign' and trying to kill them (cfr immunocompetent cell infiltrations, HLA associations, and vaccination attempts). The assumption (Vogelstein) in which we all believed some decades ago, was that 1) there is a limited number of precancerous tumour stage and we may prevent cancer if these are removed, 2) the initial cancer is local without spread and 3) if a manifest cancer is removed before spread the patient is cured/will not die. The initial observed effect of intervention (colonoscopy) did not meet the first assumption, and which was commonly agreed as the basis for shortening the intervals between colonoscopies. To our surprise, shorter intervals between colonoscopies

had no effect on – or possibly increased – colon cancer incidence in LS which is incompatible with the first assumption. And – even more surprisingly – shorter intervals between colonoscopy has not been demonstrated to reduce mortality from colon cancer, which is incompatible with the second assumption. While I do agree that there is no knowledge indicating exactly how to quantify these problems (in sum referred to as 'overdiagnosis'), it to me should be possible to estimate some parameters to make some examples (often referred to as iteration using statistical models to arrive at best fit with observations made). I do acknowledge that there are too many parameters and too heavy computational procedures to undertake Monte-Carlo-like simulations to search for strata testing all possible combinations and this is basically why the statistical models used have been developed to arrive at most probable overall results, but assuming some (extreme) parameters as a sort of sensitivity analysis to assess robustness of the results arrived at should be doable. When not done, to me, the results are 'assuming this the results are that, but we don't know to which degree the assumptions may be true and to which degree they may influence the results'.

We have added the definition of term cancer, now defined in the context of our model as "a state, in which alterations of at least three key signaling pathways or respective genes are present in one crypt". We have added a sentence to Section 1.2 of our manuscript (page 5, line 61, manuscript version with changes highlighted). For the specific setting of Lynch syndrome, the mathematical definition of the cancerous state is given in Section 4.1 (page 25, line 500): "State 3: MMR-proficient or MMR-deficient, CTNNB1 activated; APC and TP53 inactivated; KRAS activated (called cancerous)".

We fully share the reviewer's view that "cancer" neither is a synonym of "death", nor an irreversible state. However, in order to obtain measurable results and to ensure comparability with real world observations, we decided to use this operationalistic definition. In this line, the presented modeling approach is a first step towards a comprehensive understanding of Lynch syndrome-associated colorectal carcinogenesis. It is based on the basic common, current biomedical understanding with the possibility to include further biomedical concepts as soon as data and knowledge are available. The present modeling approach is deterministic, due to the complexity of the scenario acknowledged by the reviewer. Therefore, precise assessment of the robustness is only feasible through parameter variations in the present scenario, as described in Section 4.4 (page 28). We fully agree, however, that stochastic modeling naturally represents the direction to go and added these lines of thought to the Discussion (page 32, line 658).

We are well aware of alternative concepts including spontaneous disappearance of cancer (which we have integrated in the present model for pre-cancerous lesions in the revised version of the manuscript) and will pursue these questions from different angles. We agree with the reviewer that at present there is little knowledge indicating exactly how to quantify effects such as spontaneous disappearance. In this context, we would like to emphasize that the "linear model" used in the present approach only reflects the mathematical framework of linear differential equations, but does not represent the evolutionary process, which we consider as a parallel, competitive process of mutational events, persistence and regression of lesions. We further extended the paragraph in the Discussion (page 33, line 708). To enhance readability, we shortened and focused related paragraphs of the Discussion.

As an illustration of the above, the leftmost diagram in Fig 1 is not what Ahadova et al published, but in contrast to their attempt to quantify the results, the figure is indicating that the bottom pathway starting with a MMR deficient crypt is dominating. Which is compliant with results in this paper, and should lead to a re-consideration of the assumptions made (iterating). The inherent problem of probabilistic modelling, is that each argument initially (a-priori) are to be given a value (NULL is not tolerated), and the values are not to be too low (zero a-priori value will give zero as results whatever the other arguments are when calculated as a product), and a very low a-priori value will be a probability 'dark well' from which one never may escape. To escape from this, a-priori values are often arbitrarily given as 0.5 and no arguments set to lower than 0.1 (cfr InSiGHT classification system for determining pathogenicity of the variants discussed in this paper). The resulting probabilities are in consequence by definition 'false' – they are not 'real' probabilities but the consequences of the assumptions given as a requirement to use the algorithms. While the independent mutations assumed in this mns have estimatable prevalences, the complex mutations I discuss have so low a priori probabilities that they may not easily be included in probability algorithms. If these actually are the causes of cancers killing the patients, however, the main results of this paper will not be valid to explain why some patients die and others do not. Again, this is about what is meant by 'cancer' and to which degree 'cancer' may be a surrogate for 'death'. What I ask for is not an expansion in word to describe the principles in more details, but short statement(s) of results when including different a-priori assumptions in the calculations.

We thank the reviewer for highlighting the difference in illustrating the three pathways of carcinogenesis in Lynch syndrome in Ahadova et al. and in our manuscript, where the latter was not primarily with focus on quantitative results. We have now slightly changed the leftmost diagram in Fig 1, to be in concordance with the figure in Ahadova et al. and with our simulation results (Fig 11). We indeed did an analysis of different initial conditions and parameters, accounting for the influence of variants in different MMR genes (Section 4.2, page 26), the contribution of different parameters (Section 4.4, page 28), varying initial conditions accounting for Lynch-like, FAP and MSS colorectal carcinogenesis (Section 4.5, page 29). However, as outlined above, we fully agree with the reviewer's comment regarding probabilistic modeling and have added a sentence to the Discussion (page 32, line 658, see comment above).

The statement "Mutations can either activate oncogenes (called gain-of-function mutations), which normally promote appropriate cell growth and proliferation, or mutations can damage or destroy tumor suppressor genes (called loss-of-function mutations), which normally limit cell growth and proliferation." is misleading/not true: The consented view is that activation of oncogene transcription is through deactivating the normal homeostatic way these are controlled by suppressor genes through deranging the suppressor genes to loose their ability to suppress oncogene transcription. The oncogenes were denoted oncogenes by the misconception that mutations in these could increase their transcription, while the opposite is true: oncogenes loose their ability to increase cell division if mutated. To the end that mutations in suppressor genes are the main cause of gain of function of oncogenes. The normal homeostatic mechanisms activating oncogenes are intricate, and to the degree these are implicated in carcinogenesis it may be related to the inflammatory mechanisms associated to increased BMI now increasing the incidence of colorectal and endometrial cancer and which may be partly counteracted by COX

inhibitors (aspirin and naproxene). Chronic myeloid leukemia, which is not inherited, is caused by a chimeric de-novo gene caused by a somatic mutation upstream to the functional coding part deranging the genes ability to be switched off by the normally controlling suppressor-genes. Such mutations increasing the transcription of an oncogene are by and large not compatible with life and not seen in newborns. They should not be mentioned in the current context. Whatever references one may have to the opposite, the old concepts I was told in medical school were misconceptions blocking understanding both normal homeostasis and carcinogenesis. The take-home message for true gain-of-function mutations should be that they are incompatible with life and therefore not inherited. (A more detailed discussion including inherited EPCAM tail deletion causing methylation/epigenetic inactivation of MSH2 having the same effect as a deranging variant in the MSH2 coding structure is outside the level of this comment). Inherited cancer is an observed (slightly) reduced capability of de-regulating oncogenes, leading to a slightly increased probability for carcinogenesis by age. In particular, dominantly inherited cancer is incompatible with substantially increased cancer incidence in fertile ages. Which is exemplified, but not discussed, when FAP is used as an example: the probability for a single adenoma caused by the CIN pathway to become a cancer is very low, and the disease is recessively inherited because heterozygotes have very low probability to produce null-allelic cells to become adenomas which in turn have a very low probability each to become cancers. The mixture of discussing FAP and LS together, the former a recessively inherited disorder where the adenoma rarely progress to cancer and where cancer by and large emerges in the adenomas, and LS where adenomas were assumed to be rare but obligate precursers with very high risk to become cancers, has been a major confounder prohibiting the understanding of LS carcinogenesis. The current mns to me repeat this confusion, while on the contrary it should distinctly be described as a misunderstanding.

We are grateful for the opportunity to clarify these points.

Point 1: Oncogenes relevant for the development of colorectal cancer and most commonly found mutant in these tumors are *KRAS* (less commonly *NRAS* or *HRAS*) and *BRAF*. Here, specific somatic mutations cause permanent functional activation. Somatic mutations affecting codons 12 or 13 of the *RAS* genes disrupt the GTPase domain, which is functionally relevant for self limitation of the activity of the RAS proteins that typically act as mediators of growth factor-triggered signalling. Such mutant RAS proteins remain active after one initial activation event even in the absence of external growth factor signals. Similarly, mutations affecting codon 600 of the *BRAF* gene lead to a conformational change of the protein’s active center, mimicking a phosphorylated and active state that cannot be de-activated by phosphatases, which physiologically limit BRAF activity.

Our statement, which we have modified according to the reviewer’s request, now reading “Somatic mutations can either directly activate oncogenes (typically referred to in the literature as gain-of-function mutations), which physiologically promote appropriate cell growth and proliferation, through conformational changes or impairing self-inactivation” (page 6, line 69) refers to these examples.

Point 2: We completely agree that the simultaneous use of the terms dominant and recessive on the germline and the somatic level leads to confusion in the scientific community. Therefore, we would like to emphasize that the term autosomal dominantly inherited cancer syndromes, i.e., those in which one mono-allelic germline variant is associated with a phenotype of enhanced cancer risk, necessarily need to be associated with a recessive effect of the same variant on the somatic level. Otherwise, as stated by the reviewer, such conditions would not be compatible with reaching the fertile age and a sufficient reproduction rate for the condition to persist in a population. In our view, the comparison of FAP and LS, which both are autosomal dominantly inherited according to the definition, is valid, and highlights the significant differences of DNA MMR gene and *APC* gene alterations on the cellular phenotype and tumor risk.

The statements :" Lynch syndrome is caused by an inherited mismatch repair (MMR) gene mutation [4]. Colorectal cancers which develop due to Lynch syndrome therefore are MMR-deficient and show microsatellite instability (MSI) [5]." are not correct and misleading: As mentioned earlier, an inherited variant should not be denoted 'mutation', and the logical connection between the inherited variant causing cancer and the MSI phenotype is an observed association, not necessarily a logical consequence. The word 'caused' is in the present context of probability calculations misused: The inherited variant is associated with an increased probability of developing a cancer, and – actually - acquired mutations in the MLH1 genes may more frequently than inherited pathogenic variants cause MSI cancers. The major cause of an MSI cancer is not an 'inherited' variant. The concept of causation is theoretically very difficult and should be avoided in the current context: no single factor is 'the cause' of cancer – it is a coincidence of many factors. This is again implicated in the following statement: " The step-wise hypothesis has been validated subsequently in many independent studies for many different cancer types. Currently, it is expected that a minimum number of three mutation events is required to transform a normal cell into a cancer cell. This hypothesis is called the three strikes hypothesis [13].

Individuals with Lynch syndrome are predisposed to developing certain malignancies with a substantially higher lifetime risk compared to the general population. The most common Lynch syndrome manifestations are colorectal cancer (CRC, 50 % [19] compared to 6 % in the normal population) and endometrial cancer (40–60 % compared 128 to 2.6 % in women without Lynch syndrome) [4, 20]. Further, individuals have an increased lifetime risk for many other types of cancer such as in the stomach, small bowel, brain, skin, pancreas, biliary tract, ovary (only for women) and upper urinary tract [21]. Lynch syndrome carriers are predisposed to develop MSI cancers due to have an inherited pathogenic variant in one allele of the affected MMR genes MLH1 , MSH2 , MSH6 or PMS2 [18] passed down in the family from parent to child. Upon the second somatic hit inactivating the remaining allele, MMR deficiency manifests in the 136 affected cell [17]. DNA replication errors, especially those which occur at repetitive sequences (microsatellites consisting of a consecutive series of identical basepairs) cannot be corrected by the mismatch repair system. MMR deficiency leads to microsatellite instability. MMR deficiency can be an initiating or a secondary event in Lynch syndrome carcinogenesis. This is reflected by the hypothesis of three pathways responsible for colorectal carcinogenesis in Lynch syndrome [22] (see Fig 1): One pathway of carcinogenesis starts with adenoma formation, then MMR deficiency and cancer outgrowth; the second is initiated by MMR deficiency, then adenoma formation and cancer

outgrowth; and the third shows MMR deficiency as initiating event and invasive cancer growth." In this statement initially LS is considered one delineated entity, while thereafter it is declared to be four distinct syndromes caused by four different genes when deranged, and EPCAM is not mentioned as it is another gene causing epigenetic inactivation of MSH2 – the MSH2 gene is not 'mutated' when the pathogenic inherited variant is EPCAM. The four different genes have different capacity to correct replication errors in different structures in the genome. They behave differently in every demonstrated way to measure their activities and corresponding consequences when not functioning properly. It is not proper to lump them all together in one group in the current context.
In particular, it is to me unlikely that the triggering event in colon cancer associated with PMS2 is the PMS2 variant – in contrast to MLH1 and PMS2 carriers, PMS2 colon cancer may follow the CIN/APC pathway and effects of intervention indicate that these actually may be prevented by adenomectomy: in the upper leftmost part of Fig 1 PMS2-associated colon cancer may by and large follow the upper pathway. To which degree the same is true for MSH6 carriers are to me unclear. The paper is to me based on observations in carriers of pathogenic MLH1/MSH2 variants, and should indicate so both in title and text.
Reading the paper, I get the impression that a 'linear' tree-step model is commonly accepted as the ordinary carcinogenetic pathway. To me, this is not true. The most frequent cancer in women – breast cancer – and the most frequent inherited cancer in women inherited breast/ovarian cancer – is not: In contrast to colon cancer where the patient is considered cured if no spread at diagnosis and no recurrence after some years, recurrence rate for breast cancer never flattens out. In particular, BRCA1-associated breast/ovarian cancer is demonstrated to spread before a tumour is detectable – to the effect of clinical advice on prophylactic oophorectomy and mastectomy in healthy carriers.
In the three-step model assumed by the authors, in these as frequent both sporadic and inherited cancers, the three step model is now recognized as false: Often, and especially so in BRCA1 carriers, the step causing spread (which should be the biological precursor of death) is very early. The breast cancer oncologists traditionally denote it 'dormant breast cancer'. The discussion of over-diagnosis in breast cancer screening is commonly acknowledged, the corresponding in LS should be no surprise and a model to explain inherited cancer in path_MLH1/MSH2 carriers cannot ignore this. The problem with the LS discussion is that – in contrast to breast cancer screening – one assume cancer may be prevented by early diagnosis. This never was the goal of breast cancer screening, and has now been proven not to be true for LS as well. And, it is neither uniformly accepted to be true for sporadic colon cancer.
In short: The graphical image of path_MLH1/MSH2 carcinogenesis may be considered as opposing vectors in a multi-axis space, where the observed cancer incidence is the summary projection to one axis, and which is demonstrated to be a function of time between examinations to demonstrate cancer: over-diagnosis is a time-axis in the space. Without discussing this, the results presented give limited information.

We have thoroughly revised the use of terms mutation and variant, now only using the term mutation for somatic events, while referring to all germline alterations as variants.

We are also very grateful to the reviewer for discussing the concept of cause and association. In fact, the concept of multiple "causes" of cancer is inherent to our model, as germline variants only represent a modifying starting condition for the model. Therefore,

we were very happy to change the respective sentences in the manuscript as suggested by the reviewer.

Importantly, the mentioned three-pathway model is not an underlying assumption of our model. The model is based solely on the widely accepted hypothesis that multiple alterations of key signaling pathways is required for colorectal cancer formation in Lynch syndrome and beyond. In fact, the model, particularly by implementing assumptions about overdiagnosis and spontaneously disappearing lesions, shall provide information about the relative contribution of molecular events and their sequence to the overall cancer burden. These can, by implementing clinical, epidemiology, and molecular data, be aligned to clinical appearances and, as outlined in the discussion, hopefully inform about the effectiveness of specific prevention protocols in future studies.

According to the first revision, we included the aspect of over-diagnosis in the manuscript (describing the medical hypotheses and giving a model extension to account for this). However, we argued why it is at the current state of data availability not possible to fit parameters for this scenario. We also agree that considering the aspect of over-diagnosis is definitely one of the most important points for future work, as emphasized in our revised manuscript (see also comment above). We developed the modeling approach for this, given in the manuscript (Appendix A.5, page 51) which is ready to be fitted to data as soon as they are available in sufficient quality.

The above argument are to indicate why 'a linear model' is not an obvious assumption to be true, over-diagnosis when colonoscopy is a demonstrated problem, the assumption of LS colorectal cancer to be preventable by frequent enough colonoscopy is not demonstrated to be true, while – in contrast to BRCA1 associated breast/ovarian cancer – early diagnosis has been proven to dramatically reduce mortality.
The arguments above are to illustrate that while I get the impression of the paper that cancer = death validating 'cancer' as a surrogate endpoint for death, this is to me not so. And, back to the start, the paper still has no definition of 'cancer': An adenoma with micro-invasive cell growth may be biologically very different from a metastatic tumour. In short – the generalization of the concepts discussed above are to me not true, neither is the overall terminology LS when the paper describes some biological findings in in MLH1/MSH2 carriers.
Independently of the above, the assumptions: "we assume that all genes implements all mutational processes that are independent of each other, which is either due to a independence indicated by data or due to missing medical insight suggesting otherwise Independence of the Processes We require that the processes are independent of each other. In the context of the example above, we interpret from the first assumption that all combinations of mutations in the different genes are possible (i.e., there are no mutations that prevent other mutations) and that there are no additional states. This also implies that the order in which mutations are accumulated is ignored." are wrong. Mutations as events may be interrelated – in linkage terminology when estimating genetic distance between loci denoted 'inference'. The example of today is a SarsCov2 virus variant having increased infection rate due to multiple 'mutations' and the 'missing link' as an evolutionary process is not demonstrated. The evolutionary demonstration is copies of large gene sequences (transposable elements), in the current context an obvious example is the copying of the basic immune system structure to multiple close-to identical genetic

parts dispersed and specialized to have different functions as afferent and efferent pieces in the immune response, including LS cancers. These multi-topic multi-mutations events are well known in cancer, may in most cases cause cell death, but may extremely rarely cause viable cells with complex alterations in their genomes. In the very many cell divisions in MSI crypts in MLH1/MSH2 carriers, cancer may extremely rarely be caused by such events causing a viable cancer cell not to be identified and destroyed by the host immune system. Such a hypothesis is conform with current understanding of cancer and evolutionary genetics and in accordance with empirical observations in LS.

In sum, there are arguments to be discussed to interpret the conclusions. While the results may be correct, to me the mns is still not good enough discussing to which degrees the mechanisms described are the 'causes' of the cancers observed. The demonstration of the concepts in the paper to be true, is not validating that these are the major and most frequent causes neither of cancer nor of death. This is not about the algorithms and results per se, but how to discuss the assumptions and conclusions in a broader perspective. The mns would to me be more valuable and not invite the counter-arguments above if proactively discuss these in more details.

We thank the reviewer for the very thorough conceptional assessment. We have added statements to the discussion, indicating the difficulty of using molecular definitions as synonyms to clinical definitions (page 32). Moreover, we have added a sentence discussing the influence of the initial parameter settings on the representative example obtained in the simulations as well as the general limitations of deterministic modeling approaches compared to stochastic models. We also have revised terminology as suggested by the reviewer (see also comments above) and removed any unjustified referral to cause-effect relations throughout the manuscript.

In general, the design of our modeling approach, not only due to its reliance on real-world data, necessarily has to follow prevailing concepts of cancer development. However, we fully share the reviewer's concerns regarding their limitations. We hope that the revised manuscript, avoiding misconceptions, more openly and thoroughly addresses these points.

Reviewer #3:

The manuscript entitled "Mathematical Modeling of Multiple Pathways in Colorectal Carcinogenesis using Dynamical Systems with Kronecker Structure" by Haupt and colleagues has been extensively revamped and reorganized, receiving most if not all suggestion this and the other reviewers have made. The revised manuscript is definitely clearer and easier to read (for instance, there is now an explicit distinction between assumptions made for the Lynch syndrome case, and those that are general, and so on).

That said, there is still the need to double check and proof read the text, since some sentences are still a little unclear, or require revision of the punctuation. An example for instance is at page 15/55 (in the manuscript version with highlighed changes), lines 341-342: "For other types of cancer [COMMA HERE] or once new medical insights are gathered, they can and should be adapted.

We thank the reviewer for carefully reading the manuscript. We double checked the text and made slight corrections regarding clarity and punctuation, whenever needed.

Finally, I think a main point still remains to be addressed (or at least explicitly discussed): mutations and genetic alterations in cancer occur "clustered" in pathways –– NB: here not intended as sequences of events leading to carcinogenesis, but as signaling and biological pathways regulating cell processes –– so it would be interesting to understand and discuss how the model can accommodate this concept. The Wnt pathway, for instance, can be affected via alternative hits (in APC, Axin, β-catenin, etc, ...), so one is left to wonder how this is (or would be) handled in the model (if handled at all).
In other words, is the assumption that the Wnt pathway (or the MMR-deficient process, or the RAS pathway) is altered as a whole –– hence the genes in said pathways are interchangeable –– or the genes in the model are individually considered? For MMR-deficiency it is acknowledged that the mutations could be in different genes, what about the other pathways?

We fully agree with the reviewer's point that mutations in cancer occur "clustered" in pathways and added these lines of thought to the Discussion (page 32, line 682). And as there are gene-specific aspects which could indeed not be represented by a pure pathway-based model, we consider genes individually and model mutational dependencies of different genes in a general way based on existing data. For the presented example of Lynch syndrome, there was no differentiation observed in data for, e.g., the RAS pathway. As mostly *KRAS* mutations are observed in Lynch syndrome (Oliveira et al, Hum Mol Genet, 2004), we only included those mutations in the model.
For the Wnt pathway asked by the reviewer, we model *APC* and *CTNNB1* mutations with their individual consequences on cell dynamics based on current biomedical understanding and data, e.g., increased LOH event rate after APC inactivation but not after *CTNNB1* mutations, according to Ref 52, or the positive association of *MLH1* and *CTNNB1* mutations but not *MLH1* and *APC* mutations, according to Ref 25 (see model components *C* and *D*, page 19, line 369).

There are implications here, since assuming and parametrizing only using the APC gene for the whole Wnt pathway, for instance, does not exclude that when this gene is not mutated, other genes with the same effect (i.e., altering Wnt signaling) are indeed mutated. Hence the whole model might be somewhat incorrect. Given the methodological nature of the paper, this aspect could be simply discussed, not overselling the results obtained on the medical and biological side (since they could not be exactly correct...)

We strived to build our model including the currently implemented gene-specific aspects in concordance with the existing clinical and molecular data (see comment above). We agree that our model is not exhaustive and there might be other driver mutations in Lynch syndrome carcinogenesis where empirical data are scarce and thus, these mutations are currently not covered for the specific example of Lynch syndrome modeling. Due to the general structure of the model, it would be possible to consider other driver mutations in future. We added these sentences to the Discussion addressing this limitation (page 31, line 633).
